# 586. Systematic Literature Review of the 13-valent Pneumococcal Conjugate Vaccine (PCV13) Effectiveness Against Invasive Pneumococcal Disease in Children Globally

**DOI:** 10.1093/ofid/ofac492.638

**Published:** 2022-12-15

**Authors:** Johnna Perdrizet, Iwona Pustulka, Carol A Forbes, Emily Horn, Bradford D Gessner, Kyla Hayford

**Affiliations:** Pfizer Inc, New York , New York; Evidera, Krakow, Malopolskie, Poland; Evidera, Krakow, Malopolskie, Poland; Pfizer Inc, New York , New York; Pfizer Biopharma Group, Collegeville, Pennsylvania; Pfizer, Toronto, Ontario, Canada

## Abstract

**Background:**

After a decade of 13-valent pneumococcal conjugate vaccine (PCV13) use in children, many observational studies have evaluated PCV13 vaccine effectiveness (VE) in various epidemiologic and geographic settings. The objective of this study was to systematically review the global evidence on VE of PCV13 against IPD in children.

**Methods:**

A systematic literature review using Cochrane methods was conducted in November 2021 to evaluate PCV13 VE against vaccine-type and serotype-specific IPD in children. A search of Medline, Embase, the Cochrane Library (CENTRAL and CDSR), and 5 key conferences (2019-2021) was conducted, and eligible English-language articles were abstracted.

**Results:**

15 studies on PCV13 VE were included, primarily from Europe (n=7) and North America (n=3) as well as Africa (n=2), Australasia (n=2), and Asia (n=1). 11 reported VE in children who completed the full vaccination schedule [3+1 (n=3), 2+1 (n=4), 3+0 (n=3), and mixed 3+1 or 2+1 schedules (n=1)] (Table 1). 4 studies did not report VE after the complete vaccination schedule.

8 studies reported PCV13-type VE, 3 reported PCV7-type VE, and 6 reported PCV13 non-PCV7 VE. PCV13-type VE was 90 and 91% in studies using a 3+1 schedule (n=2), 76% and 79% in studies using a 2+1 schedule (n=2) and ranged from 59% to 94% in a 3+0 schedule (n=3), with variability by study design and age range (Table 1). One study reported 100% PCV13-type VE in Spain where mixed 3+1 and 2+1 schedules were used. All PCV13-type VE results were statistically significant except two outcomes restricted to children 12-24 month of age from a single study in Australia. High VE was reported against PCV13 non-PCV7 serotype IPD for fully vaccinated children across all schedules, ranging from 60% to 100% (n=6). PCV13 was effective against the shared PCV7 serotypes, ranging from 75% to 83% (n=3).

**Figure:**
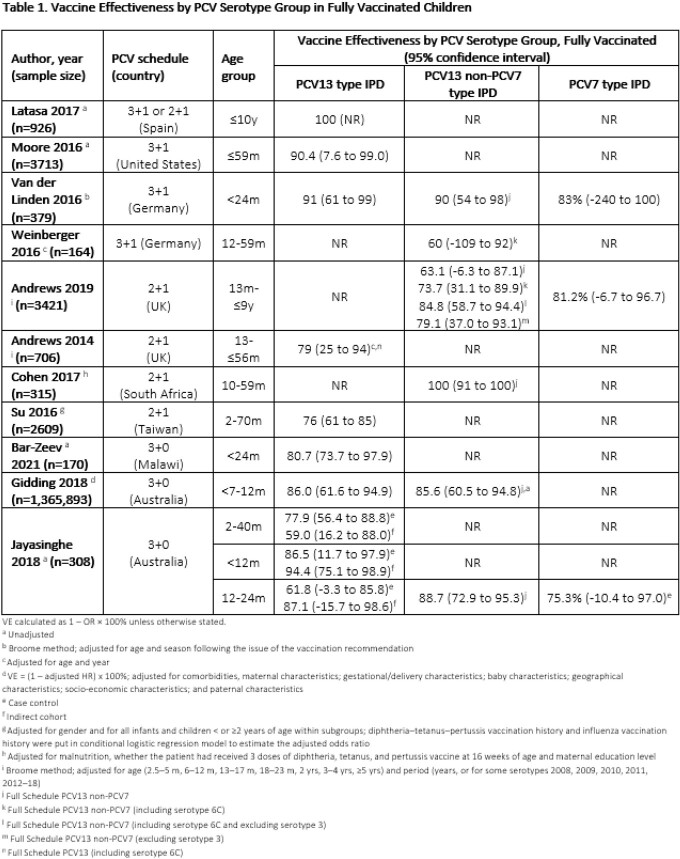

**Conclusion:**

Children fully vaccinated with PCV13 have a high level of direct protection against vaccine-type IPD, irrespective of geographic variation. PCV13 VE against the shared PCV7 serotypes remained high and PCV13 VE was also high for the 6 additional PCV13-non PCV7 serotypes. PCV13 VE tended to be higher in 3+1 schedules and additional analyses by dosing schedule are needed.

**Disclosures:**

**Johnna Perdrizet, MPH**, Pfizer Inc: Employee|Pfizer Inc: Stocks/Bonds **Iwona Pustulka, MSc**, Pfizer Inc.: I'm an employee of Evidera who have been paid by Pfizer Inc. to conduct an SLR which results are presented in the abstract **Carol A. Forbes, PhD**, Evidera (part of Thermo Fisher Scientific): Employee|Pfizer Inc: I am an employee of Evidera (part of Thermo Fisher Scientific)who have been paid by Pfizer Inc. to carry out the work reported in this abstract|Thermo Fisher Scientific: Stocks/Bonds **Emily Horn, MSc**, Pfizer Inc: Employee **Bradford D. Gessner, M.D., M.P.H.**, Pfizer Inc.: Employee|Pfizer Inc.: Stocks/Bonds **Kyla hayford, PhD**, Pfizer Vaccines: Employed|Pfizer Vaccines: Stocks/Bonds.

